# The genetic basis of inter-individual variation in recovery from traumatic brain injury

**DOI:** 10.1038/s41536-020-00114-y

**Published:** 2021-01-21

**Authors:** Daniel Cortes, Martin F. Pera

**Affiliations:** The Jackson Laboratory, Bar Harbor, ME 04660 USA

**Keywords:** Regeneration and repair in the nervous system, Neuroscience

## Abstract

Traumatic brain injury (TBI) is one of the leading causes of death among young people, and is increasingly prevalent in the aging population. Survivors of TBI face a spectrum of outcomes from short-term non-incapacitating injuries to long-lasting serious and deteriorating sequelae. TBI is a highly complex condition to treat; many variables can account for the observed heterogeneity in patient outcome. The limited success of neuroprotection strategies in the clinic has led to a new emphasis on neurorestorative approaches. In TBI, it is well recognized clinically that patients with similar lesions, age, and health status often display differences in recovery of function after injury. Despite this heterogeneity of outcomes in TBI, restorative treatment has remained generic. There is now a new emphasis on developing a personalized medicine approach in TBI, and this will require an improved understanding of how genetics impacts on long-term outcomes. Studies in animal model systems indicate clearly that the genetic background plays a role in determining the extent of recovery following an insult. A candidate gene approach in human studies has led to the identification of factors that can influence recovery. Here we review studies of the genetic basis for individual differences in functional recovery in the CNS in animals and man. The application of in vitro modeling with human cells and organoid cultures, along with whole-organism studies, will help to identify genes and networks that account for individual variation in recovery from brain injury, and will point the way towards the development of new therapeutic approaches.

## Introduction

Traumatic brain injury (TBI) is any encephalic damage caused by an external mechanical force, usually acute. The causes of such damage include automotive collisions, falls, blunt impacts, projectiles, and diverse other insults, and the mechanism and site of TBI are thus highly variable. A total of 50 million people experience TBI annually throughout the world, with the global cost to the economy estimated at $US 400 billion per annum^1^. One of the major concerns regarding TBI is its impact on families and societies. TBI is the leading cause of death in young productive people, and its incidence is increasing in the aging population^2^. TBI presents an immense challenge to the health care delivery system in low- and middle-income nations^3^. The sequelae of TBI can be long-lasting and sometimes permanent^4^; strategies that could mitigate the impairment resulting from TBI could greatly increase the quality of life and enable a faster return to productivity.

TBI can be classified in many ways according to the cause, severity, brain volume affected, and other parameters. Although the primary insult can account for the greater damage in the brain, the so-called “secondary injury” plays a vital role during the later phase of survival when other factors such as inflammation, edema, and ischemia/reperfusion, affect injury outcome. TBI causes acute disruption of cytoarchitecture, diffuse axonal injury, dendrite shearing, synapse disruption, and demyelination, sometimes increased by hematoma-induced compression, white matter loss, and further damage in following stages due to necrosis, apoptosis, and inflammation^5,6^.

It is well known that individual patients can show very different degrees of outcome and functional recovery from TBI or stroke, even when clinical variables such as the anatomical site and nature and extent of the lesion, patient age, and overall health status, are taken into consideration^7^. In TBI and stroke, because extensive clinical trials of interventions aimed at neuroprotection have not been overly successful, there is an increasing emphasis on neuro-rehabilitative therapies to enhance recovery^8^, and a new focus on a precision medicine approach to patient treatment^1^. Currently, there is little in the way of evidence-based guidelines for surgical treatment or rehabilitative interventions in TBI^9^.

The goal of applying precision medicine to enhance recovery from brain injury is challenging at present, because although outcomes following stroke or TBI are highly variable, the basis for this variation is largely unknown. Animal studies, discussed below, have clearly demonstrated the impact of genetics on recovery following injury to the CNS. While a number of clinical studies have attempted to understand how genetics influences recovery from brain injury in the human, this field is still at a very early stage. Recovery from TBI represents a very complex phenotype. A limitation of much work to date has been a reliance on candidate gene studies with limited numbers of patients, some of which have not proven reproducible^10,11^. The Genetics Association in Neurotrauma (GAIN) Consortium (https://intbir.nih.gov/node/45) will produce the first GWAS to study biological mechanisms that modulate response and recovery after TBI. Only recently have large scale GWAS studies reported on recovery after stroke^12,13^. These studies are illustrative of the challenges facing the field, because they have identified candidate genes whose relevance to post-injury recovery is unclear. The validation of these candidates will require new approaches using human cells in vitro and whole-organism studies in model systems.

Although recovery from injury to the CNS takes place in a very different environment and under very different circumstances to brain development, many developmental processes, including neurogenesis, axon sprouting and elongation, synaptogenesis, and synaptic remodeling, are all critical to restoration of function in TBI in the postnatal CNS^14^. Indeed, many genes implicated in neural recovery in animal and human studies have essential roles in brain development. Over the past several years, the application of next-generation sequencing technologies to the study of developmental disorders has resulted in the identification of many novel genes that have important roles in human brain development^15–17^. It is reasonable to expect that variants in these neurodevelopmental genes will influence brain repair in the adult. It is also clear that apparently normal individuals may carry mutations in such genes, and that the effect of a developmental mutation in such an individual might be unmasked following brain injury, because the environment in the adult is not as supportive of neurogenesis or plasticity as that in embryonic or fetal life.

This review considers the genetics of recovery from TBI from a developmental standpoint. We include some informative studies on spinal cord injury. Although the pathobiology of TBI is quite different to injury in stroke, there are some overlaps in recovery mechanisms, so we consider some relevant studies in stroke as well. In this broad survey of the field, we refer to previous reviews and meta-analyses of particular topics where appropriate, and the citation of primary literature is focused on recent data and areas that we have chosen to highlight.

## Neurobiological basis of recovery from CNS injury

Injury caused by trauma or stroke sets in motion a number of processes that have the potential to repair or circumvent the damage caused by the injury^18,19^. For example, the sensory and motor cortex can undergo remapping to transfer function to unaffected areas. Regrowth of fibers from the side contralateral to a lesion can contribute to movement on the ipsilateral side following injury. Behavioral activity interacts with these repair processes, and activity- and experience-dependent plasticity are important to functional recovery. At the cellular level, recovery mediated by the remapping of neural circuits requires axon sprouting, outgrowth, spine morphogenesis, synaptogenesis, and synaptic pruning. Neurogenesis can enhance recovery in model organisms^20^ and may play a role in humans as well, though the extent to which neurogenesis continues during adult life in primates remains controversial (below). Whatever the role of neurogenesis in the adult human, it is apparent that the overall capacity for remodeling and plasticity diminishes with aging^21^.

Axonal sprouting is fundamental to the rewiring that must occur to bypass damage within circuits. Sprouting can forge new connections around the lesion and on the contralateral side. Axonal sprouting cannot be measured clinically, but primate studies have established that it is part of the post-injury response in monkeys^22^. Myelinated fiber tracts may also undergo injury and subsequent incomplete repair^23^. The recovery process invokes many biological strategies involved in learning and memory, including long term potentiation and dendrite formation^24^. Tonic (extrasynaptic) GABA inhibitory activity can be reversed to enhance recovery. AMPA receptors stimulate BDNF release and enhance learning and memory post-injury. Enhancing plasticity early on may worsen outcomes, but later on, will enhance recovery. Oligodendrocyte precursor cell activation may promote remyelination.

These repair phenomena occur in an adult brain environment that is hostile to rewiring, because it expresses inhibitors of axon regeneration, including myelin-associated proteins, chondroitin sulphate proteoglycans, and guidance molecules. Myelin associated proteins and chondroitin sulphate proteoglycans constrain axon outgrowth, and guidance molecules limit axonal growth cone activity. Experimentally, interfering with of these inhibitory pathways can have profound effects on outcome^25^. The regenerative niche is transient and unique to the injured brain. Recent studies suggest that widespread rejuvenation of the adult CNS environment through cell therapy can promote repair processes. In spinal cord injury, transplantation of embryonic neural progenitor cells can overcome limitations of axonal growth to establish neural bridges across an injury zone^26,27^. Engraftment of neural progenitor cells in this model results in prolonged maintenance of a regenerative transcriptome in host neurons that resembles a reversion to an embryonic state^28^.

In animal models, expression of neurotrophic and growth factors is increased after injury, accelerating neurogenesis^20,29^. Neurogenesis has the potential to replace damaged cells, but can also provide for enhanced recovery through paracrine effects, because neuroblasts, in addition to their potential for replacing damaged cells, can promote a remodeling environment. It has been demonstrated that interference with hippocampal neurogenesis will impair functional recovery after TBI in some experimental paradigms^30–32^.

Whether or not neurogenesis takes place in the adult human is currently controversial. Some studies have failed to demonstrate proliferating cells in the adult CNS, whilst others have demonstrated their presence in an equally convincing fashion, and the basis for the discrepancies in these studies remain unresolved^33–36^. Whatever its role in the adult, neurogenesis is almost certainly relevant to repair of injury or ischemic damage in the neonatal or pediatric context, and it may be that quiescent stem cells exist in humans that may be called into action in the face of injury. In mice, aging brains have quiescent stem cells kept in a dormant state by inflammatory signals and antagonism of the Wnt pathway^37,38^. Nonetheless, these cells can be activated by injury.

Microglia play an active role in recovery from damage to the CNS including TBI^39^. These innate immune cells can participate in clearance of debris after injury, remodeling, neurogenesis, angiogenesis, oligodendrogenesis, and remyelination. However, microglia can also have a detrimental effect, through the production of inflammatory or neurotoxic cytokines. Polarization of microglia has been well documented, and as in other tissues, a generalization is that M2-like cells play a role in all repair processes. Recently Willis et al. showed that enhancing turnover of microglia either through genetic depletion and replacement or pharmacologic manipulation considerably enhanced recovery after TBI, through increased neurogenesis mediated by an IL6 response^40^.

## Brain repair and developmental pathways

**Fig. 1:**
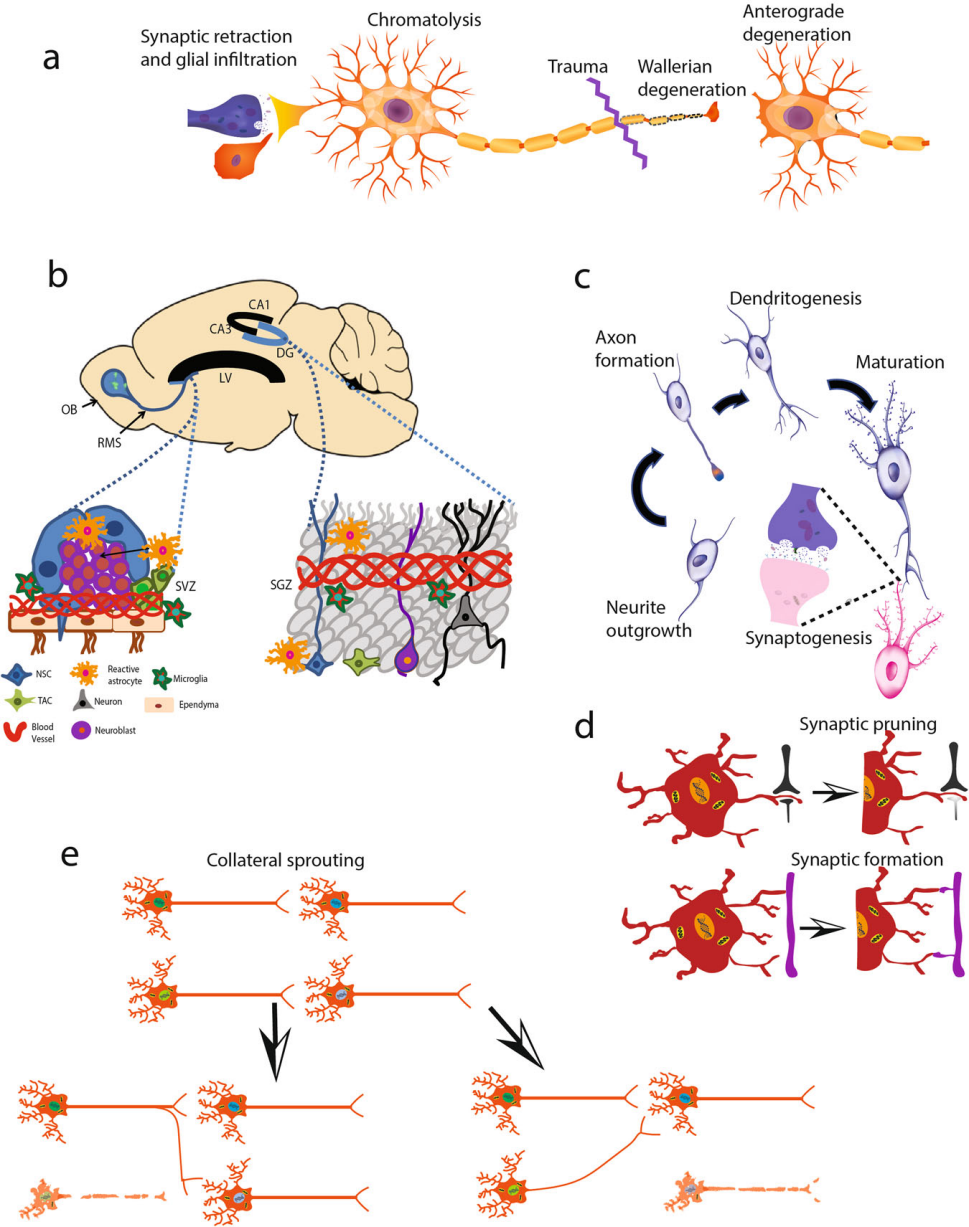
Damage and Repair at the Cellular Level in the Central Nervous System. **A** Traumatic injury through shearing or crushing action leads to Wallerian degeneration distal to the site of injury and accompanying demyelination; retrograde degeneration leads to chromatolysis with swelling of the cell body, nuclear displacement, fragmentation of the rough endoplasmic reticulum and metabolic changes. Synapses withdraw, and glial infiltration and proliferation ensue. These effects on synapses can affect neurons in circuits either upstream or downstream from the injured cell. **B** Neurogenesis is enhanced by injury in animal model systems in the subventricular zone and in the hippocampus. The extent to which neurogenesis occurs in the adult human is unclear, though it is known in model systems that quiescent neural stem cells can undergo activation in the adult. Glia and endothelial cells can modulate neurogenesis. **C** Connectivity may be restored by outgrowth of neurites, axon formation, dendritogenesis, and synaptogenesis. In the CNS, these processes may be limited by a non-permissive environment. **D** Synaptic pruning refines and strengthens connections. **E** Collateral sprouting can find new routes in spinal tracts to avoid a lesion zone. *NSC* neural stem cells, *NPC* neural precursor cells, *TAC* transient amplification cell.

Many developmental pathways are involved in the repair of injury to the CNS; neurogenesis, axonal sprouting, and growth factor dependence characterize both development and regeneration. There are clear differences between axon sprouting in development and in repair in the adult CNS. The axon sprouting that occurs in response to stroke is associated with a different transcriptome than that seen during development, and it changes with age^41^. However, a number of key developmental regulators are known to function in repair in the adult CNS (Fig. 1).

Neuroplasticity is key to repair in the CNS. In searching for sources of genetic variation in CNS repair capacity, it is worth considering new findings regarding neurodevelopmental genes. Human studies have shown that disruption of processes involved in neuronal plasticity are characteristic of childhood neurological disorders caused by mutations in neurodevelopmental genes. MECP2, FMR1, TSC1 and 2, UBE3A, and NF1 all affect dendritic spines and synapse morphology. TOR1A influences synaptogenesis in the cerebellum, and SHANK3 modulates the expression of receptors for AMPA and NMDA and in turn impacts on plasticity, specifically long-term potentiation. ATXR is a developmental gene that controls neuron survival and migration, and assists in growth cone formation. A survey of gene networks impacted by developmental disorders shows that chromatin remodeling, cell proliferation and migration, synaptic networks, and long-term potentiation are often targeted in autism spectrum disorders^16^. All of these processes are critical to plasticity.

Deleterious variants in neurodevelopmental genes could greatly impact brain repair after TBI. Copy number variations and single nucleotide variants that are associated with neurodevelopmental disorders often show variable penetrance or expressivity, and individual phenotypes are influenced by genetic background and modifier effects^42^. It is easy to imagine that patients bearing such mutations who do not show a developmental phenotype might be impaired in recovery processes in the adult, where the environment is less favorable to plasticity. These variants would be relatively rare and would be expected to have intermediate effect, characteristics that would hamper their discovery through GWAS.

## Genetic studies in mice

There are three lines of evidence from studies in mice that show the impact of genetics on the recovery from damage to the CNS. First, some reports have identified differences between mouse strains in recovery in various models of brain injury. Second, reverse genetic studies that interrogate the effects of deletion of candidate genes on CNS recovery have provided evidence for positive and negative regulation of neural repair. Finally, a few forward genetic screens have been carried out to identify new loci involved in the CNS recovery response. We discuss some examples of all three types of investigation^43^.

There is evidence for strain-dependent differences in inherent capacity for functional recovery after CNS injury, with some findings highlighting axon growth and the inflammatory response in mediating the recovery process. A study of four strains given contusion injury to the spinal cord showed better recovery of function in C57Bl/10 and B10.Pl mice relative to C57Bl/6 or BALB/c^44^. In another study of a spinal cord injury model, better axonal growth into the lesion area was observed in 129 × 1/SvJ mice, and this was associated with a decreased chronic inflammatory response relative to C57Bl/6^45^. There were fewer macrophages in the lesion of 129 × 1/SvJ animals, more neurons and astrocytes, increased levels of laminin, and lower levels of CSPG. A third spinal cord injury reported that 129 × 1/SvJ mice displayed better corticospinal axon extension relative to C57Bl/6^25^. Axon regeneration was enhanced in both strains on a Nogo−/− background. The strain differences were reflected in an in vitro study of dorsal route ganglia neurite outgrowth. Again, more macrophages persisted in the lesions of the C57Bl/6 animals. Differentially expressed genes in the two strains were associated with neurite growth, synapse formation, inflammation, and immune response. A recent study on oxidative stress in rat neuronal cultures revealed strain differences in the innate neuronal response that might reflect the ability to adapt to an inflammatory environment^46^.

Much previous work has addressed the effect of gene knockouts on neuroprotection or sensitization of the mouse brain to injury. A more limited number of experiments in mice have examined the effect of knockouts of specific genes on recovery from CNS injury. Many of these have focused on extracellular signaling molecules or their receptors. For example, studies have shown that genetic ablation of factors that block neurite extension can enhance recovery. Mice deficient in Nogo A B or C, myelin enriched inhibitors of neurite outgrowth, show enhanced regeneration of the corticospinal tract following SCI^25^. Knockdown of Epha4, a widely expressed ephrin receptor that is an inhibitor of growth cones, enhanced regrowth of descending axons after SCI and had a similar effect in stroke models^47^. Inhibition of its downstream target, Rho-associated kinase had a similar effect.

The capacity for axonal regeneration declines with age in mice, and at least part of this decline is associated with an age-related decline in the MTOR pathway driven by the negative MTOR regulator Pten. Pten is strongly inhibitory to axonal sprouting, and deletion of this gene can enhance sprouting and recovery from CNS injury. Pten and Socs3 knockout improved sprouting of corticospinal axons and recovery of limb motor control^48^. Conditional deletion of Pten after spinal cord injury enhanced recovery and accelerated axon outgrowth^49^. The effect of Pten deletion on regrowth of corticospinal axons persisted for up to one year following an injury^50^. However, Pten deletion in older mice was less effective in enabling axonal regeneration, perhaps due to increased microglia and astrocyte activation in the aged animals^21^. In another study of aging and the regeneration, administration of Osteopontin to adult mice enhanced IGF1 responsiveness in an axon sprouting assay back to levels seen in young animals, and the combination promoted regrowth of the corticospinal tract^51^.

GDF10 is a TGF beta superfamily member that is induced in the peri-infarct region in stroke. This growth factor has been reported to promote neural outgrowth and functional recovery in stroke models. GDF10 induced a unique stroke transcriptome that differed from postnatal (P4) developing brain^52^. GDF10 administration increased axonal sprouting in the adult and enhanced functional recovery from stroke. This effect of GDF 10 was mediated through down regulation of Pten and upregulation of axonal guidance molecules.

Inhibition of CCR5 receptor signaling was shown to enhance learning, memory, and plasticity in the hippocampus and cortex^53^. Knockout of this gene was associated with faster recovery of motor control in a stroke model and better recovery of cognitive function in a TBI model^54^. The effect could be phenocopied by the chemical knockdown of CCR5 signaling. Deletion of CCR5 preserved dendritic spines, and established new projections to the contralateral cortex. In a human study, Joy et al. exploited polymorphisms in this gene to show that a loss of function was associated with faster recovery from stroke^54^.

These and other studies of the role of specific genes in recovery from CNS damage are described in Table 1. For each study, the table lists the biological processes most affected by the gene in question, the functional outcome assessed, and the underlying changes at the cellular level. It is important to distinguish these studies from ones that are designed to discover naturally occurring genetic variants that affect recovery from injury, such as those focused on strain differences discussed above. It is also important to remember that gene deletion studies performed in one strain may be subject to strong genetic modifier effects, such that the response of different strains to gene deletion may be quite divergent.

**Table 1:** Studies of the influence of specific genes on recovery in mouse models of traumatic brain injury, spinal cord injury, and stroke.

Biological Process	TBI Model	Gene	Functional assessment	Cellular mechanism	Reference
Cell death and inflammation	Lateral fluid percussion	BDNF Val66Met v wild type	Administration of BDNF to Val66Met carriers improved motor and cognitive function	↓apoptosis, inflammation, and gliosis in wt mice	^85^
	CCI	Ccr2	Better locomotor recovery in -/- mice	↓macrophage infiltration ↑neuronal survival in CA1-CA3 of hippocampus in -/- mice	^86^
	CCI	Il13	sensorimotor function enhanced by IL13 administration	↓pro-inflammatory macrophage infiltration, ↑phagocytosis of dead neurons	^87^
Neurogenesis	CCI	Epo	Epo administration improved sensorimotor function and spatial learning	↑neurogenesis in mice given Epo	^88^
	CCI	Gdf5	Improved cognitive function and decreased behavioral dysfunction	↑neurogenesis in mice given Gdf5	^89^
	Cryogenic lesion, CCI	Hrh1 postsynaptic and Hrh3 presynaptic histamine receptors	Improved sensorimotor function in Hrh3 -/- mice or mice given H3R antagonist	↑neurogenesis and recovery in H3-/- mice or mice given H3 antagonist ↓neurogenesis in Hrh1-/- better neuronal differentiation in wt mice	^90^
Axonal sprouting	SCI	Ngr1	Histological assessment of axonal regeneration	NogoA -/- shows greater regenerating fibers	^25^
	Unilateral pyramidotomy	Pten-Socs3	Pten-Socs3 deletion improves motor function	Increased sprouting of uninjured axons to denervated cord in KO animals	^48^
	SCI	Pten	Improved motor function in conditional KO mice	Increased regenerative growth of corticospinal axons	^49^
	Photothrombotic stroke	Gdf10	GDF10 administration improved and knockdown worsened motor recovery	Increased axonal sprouting in mice given GDF10, with downregulation of PTEN	^52^
Dendritic spine remodeling	CCI	MMP9	Histologic assessment only	No ↓in spine density or spine shrinkage following TBI in MMP9-/- mice	^91^
	CCI	Rhoa-Rock1	Rho conditional knockout in postnatal neurons or ROCK chemical inhibition improved motor and cognitive recovery	Rho inhibition reduces pathological spine remodeling and loss post-TBI	^92^
Myelin regeneration	Contusion/compression SCI	PAR2	Histological assessment of myelination post-injury	Improved myelin resiliency and remyelination in PAR2 -/- mice	^93^
	CCI	Plat	-/- mice show greater sensorimotor defects and reduced spatial learning and memory, decreased axonal conduction rate	Administration of TPA to -/- mice enhanced white matter structure, increased compensatory neuronal sprouting	^94^
Revascularization	Cryogenic lesion	Il6	Pathologic assessment of lesion	↓vascularization, poor recovery in IL6 -/- mice	^95^
General recovery	photothrombotic stroke	Epha4	Motor function recovery enhanced in Epha4 partial knockdown mice and by downstream inhibition of pathway with Rho kinase inhbitor	Not assessed	^47^
	Photothrombotic stroke, CCI	Ccr5	Enhancement of motor function recovery in stroke and cognitive ability in TBI after shRNA or chemical knockdown of CCR5	Preservation of dendritic spines and new cortical projections to contralateral cortex in stroke	^54^

*CCI* controlled cortical impact, *SCI* spinal cord injury, *wt* wild type, *KO* Knockout.

In the past, most studies of recovery in the CNS in mice have relied on a few inbred strains. Mouse genetic diversity panels provide for a much broader interrogation of the effect of genetic background on recovery and for improved modeling of human disease. In a study of the eight founder strains of the Collaborative Cross^55^, dorsal root ganglion neurons from CAST/EiJ (derived from a strain of wild mice) showed the highest capacity for axonal growth on an inhibitory matrix of CNS myelin in vitro. In three in vivo models of CNS injury, dorsal root ganglion regrowth, optic nerve injury, and ischemic stroke, CAST/EiJ showed better axonal outgrowth. Further investigation of gene expression in strains with high medium and low capacity for outgrowth showed that the differences were mediated by the degree of induction of inhibin/Activin A. A follow-up study also found an up-regulation of Ascl1 due to a down-regulation of miR-7048-3p in Cast/Ei, a phenotype that increased neurite outgrowth in the dorsal root ganglion after axonal injury^56^. Loss and gain of function experiments in vitro and in vivo confirmed this activity.

## Human studies

The advantage of working with model systems include uniformity of age, sex and genetic background, well-controlled reproducible injuries, controlled environment, and quantitative uniform endpoints. There are many challenges to clinical studies of recovery from TBI, including the size and diversity of the patient cohorts, heterogeneity of injuries, variations in post-injury environment, care and management, a diversity of endpoints analyzed, and wide variations in the time of follow-up. Most studies report medium- or longer-term outcomes. The Glasgow Outcome Score (GOS) is a widely though not universally used metric. Recovery from injury to the CNS is not a simple endpoint and clinical outcomes could be influenced by a diversity of biological and pathological processes.

Most of the human studies reported to date are retrospective and were centered on candidate genes that are either hypothesized to influence recovery after TBI or are involved in related pathological processes. Important selection biases in these studies relate to gender, race, and ethnicity. It is known that males tend to participate in hazardous activities more often than women, such that on average in TBI studies, only one-third of the patients are females. Genetic studies of TBI outcome in humans, like many studies of genetic association with disease, have been biased towards inclusion of Caucasian individuals. It is important to overcome this limitation for scientific reasons^57^, and to ensure equitable access to innovations in health care. Here we focus on a few of the most widely studied genetic polymorphisms and thereafter discuss several very recent GWAS analyses of recovery from stroke. A summary of results from work on the more widely studied candidate genes is provided in Supplementary Table 1. These studies illustrate the challenges in reaching general conclusions from such studies, owing to variability in outcome measures, clinical endpoints, mode of assessment, and subject characteristics.

## Brain-derived neurotrophic factor (BDNF)

BDNF belongs to the neurotrophin family of growth factors and exerts its actions through binding to TrkB and the p75 receptor. BDNF is widely expressed in the central nervous system (CNS), and it plays pleiotropic roles in a number of processes during development and adult life, including neurogenesis, glutamatergic and GABaergic signaling, neuritogenesis, and long-term potentiation. This neurotrophin has also been one of the most widely studied genes in the context of TBI and stroke, because of the existence of a genetic variant with known functional consequences. A common single nucleotide polymorphism (SNP) within the coding region of BDNF gene found in 30–50% of the population results in the substitution of valine (Val) with methionine (Met) at codon 66 in the prodomain of the protein (Val66Met). The amino acid variant interferes with the sorting and secretion of the factor, resulting in a decrease in its activity-dependent release, which impacts on all downstream processes modulated by this factor. This phenotype diminishes BDNF protein secretion and growth cone retraction, critical to axon extension^58^. The role of BDNF and its polymorphisms is one of the most intriguing examples of the potential impact of genetics on the CNS in health and disease. In physiological conditions, the 66met allele has been associated with lower cognitive performance^59^, and less gray matter volume, and it has been implicated in pathologies such as Alzheimer’s disease^60^ as well.

However, the role of BDNF in recovery from TBI or stroke has been controversial. A meta-analysis in stroke patients found the Val66Met phenotype to be predictive of poor outcome with an Odds Ratio (OR) of 2.60^61^. In another study, Met-carriers were reported to show poor recovery after stroke but not TBI^62^. In fact, several studies carried out on war veterans after decades post-TBI showed that Met variant of rs6265 BDNF has *better* preservation of general intelligence and executive functioning after prefrontal cortex (PFC) damage^63^. In another report, BDNF was found to be the second most consistent predictor of cognitive status after TBI, just after intelligence prior to injury^64^ (known as the best predictor for recovery). The rs7124442 and rs1519480 SNPs were also found to be associated with post-injury recovery of general intelligence on combat veterans with focal penetrating TBI after 15 years of the event. In a pediatric study^65^, patients with the rs6265 variant had a better behavioral outcome at 6 months post-TBI. However, that difference disappeared at 18 months.

As noted above, Val66 carriers have been shown to perform better in cognition and to show better outcomes in neurodegenerative diseases compared to Val66Met individuals. The explanation of these differences in some of these studies might reside in the area impacted by the lesion, the different effects of pro-BDNF and BDNF, and changes in p75NTR and TrkB expression. Not only BDNF but pro-BDNF can be secreted, and the two forms show different binding affinities to their receptors. The high-affinity receptor TrkB binds BDNF preferentially, and the low-affinity receptor p75NTR binds pro-BDNF. Binding to Trkb is associated with pro-survival actions, but binding to p75NTR is pro-apoptotic. The expression levels of both forms of BDNF and pro-BDNF, as well as TrkB and p75NTR, can change their expression ratio during life and neurological illnesses. These complexities may account for the disparate outcomes observed.

Advanced age is well-known to impact negatively on TBI outcome. Failla et al.^66^ examined the rs6265r (Val66Met) and rs7124442 SNP (T > C, which impairs trafficking of the BDNF transcript) of the BDNF gene in the context of TBI in patients of different ages. It was found that these polymorphisms interact with age and modify mortality rate after severe TBI. During the acute phase, the anticipated low-risk group (predicted to be Val and 7124442 C) had the lowest survival rate. In the post-acute phase and as expected, the low-risk phenotype had the highest survival rate. However, this was not true for older patients. It seems that during aging the BDNF signaling and its response against injury might change due to SNPs within the gene itself, the BDNF receptors, or any other transcriptional factor associated with the signaling pathway.

Severe TBI can induce coma and the vegetative state. Since BDNF is present in several brain areas, participates in many neural activities, and its polymorphisms are associated with functional variations in neuronal activity; it is reasonable to hypothesize that genetic variation at this locus might influence recovery from vegetative state. Nonetheless, a homogenous sample of patients with this condition after TBI and with val66met variation did not show differences in recovery of consciousness or cognitive improvement^67^. An explanation for the absence of association could be caused by a “point of no return” due to extensive and irreparable damage in the brain.

The variability in the outcomes of these studies thus highlights the likely interaction of genes, environment, and age with the nature of injury and the endpoints under study. Thus, the studies above need to be interpreted cautiously. Future research will have to use polygenic approaches, next generation sequencing, gene-set enrichment analysis, and, machine-learning-assisted analysis, to finally unravel the true relation of BDNF polymorphisms and TBI.

## Apolipoprotein E (ApoE)

Apolipoprotein E protein is a principal constituent of blood lipoprotein transporter. There are three main alleles of APOE, 2, 3, and 4, 3 being the most common one. The ApoE4 allele has been associated with Alzheimer’s Disease, amyloid deposition, impaired cognition, and other neurological conditions, including recovery from TBI (review),^68^. The basis for the widespread involvement of this ApoE allele in diverse disorders of the CNS is not clear.

It has been difficult to reach firm conclusions regarding the impact of APOE genotype on recovery from TBI; the size of the patient cohorts, patient age, the type and extent of lesion, clinical endpoints, and statistical analysis has been quite variable across many studies, and undoubtedly these factors have confounded the interpretation of the data. The APOE genotype can also impact on co-morbidities which may differ from global outcomes.

Nevertheless, meta-analyses of the effect of APOE4 status point towards a role in recovery, while illustrating how additional variables can affect the outcome. A meta-analysis conducted in 2015^69^ indicated that the adverse effects of APOE4 carrier status were more pronounced in pediatric TBI than in adults. The OR for a poor outcome of childhood TBI in E4 allele carriers relative to the remaining population-averaged 2.36 across the studies. A second meta-analysis also reported in 2015^70^ began with 42 potentially eligible studies but excluded 30, mostly on the grounds that they did not use standard endpoints. A significant effect was found for long-term (>6 months) outcomes only with E4 carriers showing increased risk of a poor outcome with an overall OR around 1.4. A meta-analysis published in 2019^71^ found a worse outcome in APOE4 carriers with an OR of 1.39. Finally, one meta-analysis^72^ found no correlation between APOE4 genotype and cognitive outcome following TBI.

The difficulties are illustrated by consideration of a few individual studies. Moran and colleagues studied a cohort of children, and they could not find any differences other than a lower GCS (Glasgow Coma Scale) in APOE4 carriers^73^. Ponsford and colleagues^74^ only found an association between ApoE4 and worst GOSE (Glasgow Outcome Score), especially among women. However, they could not find lower GCS or post-traumatic amnesia (PTA) associated with ApoE4. Merritt et al. found that the ApoE4 genotype was associated with high levels of psychiatric distress in veterans^75^. Interestingly, the lack of association of ApoE4 genotype with outcomes has been shown in different populations such as African, British, and Indian^76–78^. Future studies will need to look closely at how genetic background modulates the effect of ApoE4 genotype of individual response, as recently noted in a study in mice^79^.

## Dopaminergic system

The dopaminergic system plays a variety of roles in the CNS including the regulation of mood, cognition, behavior, reward, and executive function involving pathways such as the mesocortical and mesolimbic tracts. Recent studies have addressed the effects of polymorphisms in dopaminergic-associated genes such as dopamine receptor 2 (DRD2), Ankyrin repeated kinase domain containing 1 (ANKK1), Solute carrier family 18 member A2 (SLC18A2), dopamine transporter (DAT) and Catechol-O-methyl transferase (COMT) in TBI.

DRD2 is a G protein-coupled receptor that is involved in memory formation and synaptic plasticity, and it is a crucial receptor of many antipsychotic drugs. ANKK1 is serine/threonine kinase found in astrocytes and plays a considerable role in modulating dopaminergic reward processes. ANKK1 is also closely linked to DRD2 expression as rs1800497 polymorphism with a single T allele reduces DRD2 expression in the ventral striatum by 30–40%. Failla et al. found that SNPs in two adjacent genes, ANKK1 rs1800497 and DRD2 rs6279 were associated with cognitive outcome after severe-TBI at 6-months^80^. However, at 12-months only ANKK1 remained significant, and after adjusting for multiple testing no significant associations could be maintained. The combination of two prospective multicenter studies analyzing the TBI outcome at six months concerning cognitive function showed that rs1800497 T/T patients had the worst outcome compared to C/T and C/C patients^81^.

Recently, Treble-Barna and colleagues^82^ analyzed a control group vs. a TBI cohort of children and followed them up to seven years. A total of 32 SNPs were analyzed across different dopamine-related genes. Genetic variation within SLC18A2, including rs464040 and rs460000 were associated with short- (6 months) and long-term (up to seven years) impairment following injury. In the case of ANKK1 rs1800497 and rs2734849, an association was found only for short-term impairment. Behavioral deficits in short- and long-term memory exhibited interactions with SLC18A2 rs464049, long-term deficits with rs1042098, and executive deficiencies within the short-time period with rs464049 and rs460000. In the same study there was no association with outcome and COMT and DRD2 genotypes; however, the rs1800497 SNP in ANKK1 gene exhibited high interaction, with the poorest outcome in TBI in children. As noted above, it is known that ANKK1 is closely related to DRD2 and polymorphisms in the former can affect the latter; actually, people with this polymorphism display lower dopamine receptor density, hence lower ligand binding. The rs6277 allele which is a *C947T* polymorphism within the *DRD2* gene was analyzed in a Caucasian sample of TBI patients and followed up to 6 months^81^. These researchers found that this SNP confers verbal learning improvement without mental flexibility or processing speed. The result suggests that this receptor’s effects are not global but limited to areas where it is highly expressed such as basal ganglia. Projections from this area connect to memory- and learning-related regions in subcortical prefrontal and hippocampal zones.

A meta-analysis of DRD2, ANKK1, SLC18A2, and COMT polymorphisms assessed which of the SNPs actually influence the expression abundance activity or affinity of the cognate protein product^83^. DRD2 splicing was affected by the rs1076560 variant, and ligand binding of DRD2 was affected by the rs1800497 polymorphism in ANKK1. The Val158met polymorphism in COMT had marked effects on the abundance and stability of the protein and on its enzymatic activity. Analyses of this nature are very helpful in prioritizing variants for further study. Thus, Nekrosius et al. found an association of increased risk of delirium after TBI in patients with the rs4680 mutation (Val158Met^84^).

## Variants in other pathways

Variants in a number of other genes have been reported (generally in modest-sized studies) to impact on the recovery from TBI in patient studies. These are summarized in Supplementary Table 2. Some of the pathways that have been investigated in multiple studies include glutamatergic transmission, purinergic transmission, immunomodulation, and solute and water transport. As with the clinical studies discussed above, there is great diversity in outcome measures, clinical endpoints, mode of assessment, and subject characteristics. Single studies of common polymorphisms with limited follow-up have limitations, but the outcomes of these works may hold clues for future studies.

## Recent Gwas studies of functional outcome of stroke

Two recent GWAS studies have identified candidate genes for the determination of functional outcome after stroke. The GISCOME meta-analysis study^12^ assessed over six thousand patients using a modified Rankin Scale that evaluates the extent of disability at a 60–190 day time point. Interestingly, the strongest variant was in a trans-QTL locus controlling PPIR21, a regulatory subunit of protein phosphatase 1 involved in neural plasticity, similar to PPP3CC (Supplementary Table 2). Other possible variants identified were NTN4 (netrin 4) an axon guidance molecule, TEK (a protein kinase receptor for angiopoietin 1), and PTCH1 (Hedgehog receptor). The identification of these molecules leads to testable hypotheses regarding their function in brain repair, through neuronal or vasculature processes. A second GWAS meta-analysis^13^ included a discovery, replication, and joint phase to study associations with recovery measured again on the Rankin Scale. This study identified PATJ, a gene encoding an epithelial tight junction protein. The relationship of this gene to biological recovery post-stroke is uncertain. As with other GWAS studies, epigenetics and the environment will interact with clinical variables to determine the association of a variant with recovery from brain injury, and the association is not proof of causality. Additional analysis of the epigenome, transcriptome, and proteome will further refine conclusions from large-scale studies. However, the application of GWAS to recovery from TBI represents an unbiased approach to identify new genes and pathways that can be subjected to functional analysis through in vitro testing and in animal models.

## Conclusions and future directions

Recovery from brain injury invokes a number of processes involved in neurodevelopment. We do not yet fully understand how these processes operate in the context of injury in the adult organism. The activities of the innate and acquired immune systems are a key difference between the two. Nonetheless, emerging human genetic data on the function of neurodevelopmental genes in plasticity will inform studies of gene network function in adult brain repair. Studies in mice provide strong evidence for a genetic basis of strain differences in the recovery from TBI or stroke, and have identified a number of genes that clearly influence key processes in recovery. However, most of these genes (with the exception of CCR5) have not been identified in human studies of interindividual variation in recovery. There are many challenges to examining the genetic basis of recovery from brain injury in humans, and the results of many studies performed to date are not really conclusive. The evidence suggests that BDNF and APOE loci are involved in recovery from TBI or stroke, but recent GWAS studies in stroke recovery have failed to implicate either. Indeed, the recent GWAS studies have brought forward new candidates but these will require validation and further analysis.

The application of unbiased genetic approaches to identify genes and gene networks involved in recovery from CNS damage has just begun. A coordinated approach using human and mouse cell-based screens, along with whole-organism studies in mice, will enable rigorous assessment of candidate regulatory factors and networks, and provide new targets for pharmacologic or cellular interventions to enhance recovery. These components, in conjunction with careful and adequately powered clinical studies, will help to enable a precision medicine approach to neurorehabilitation and repair.

## Supplementary information

Supplemental Material
