# Supplementary material for: The genetic basis of inter-individual variation in recovery from traumatic brain injury
Source: NPJ Regen Med. 2021 Jan 21;6:5. doi: 10.1038/s41536-020-00114-y (PMC7820607; doi:10.1038/s41536-020-00114-y)
Supplement: Supplementary file 1 — Supplemental Material [file 41536_2020_114_MOESM1_ESM.pdf]

GENE	SNP	AUTHOR	OUTCOME	FUNCTION ANALYZED	TBI GRADE (GCS)	NATURE OF LESION	ASSESMENT	TYPE OF STUDY	TIMEFRAME OF EVALUATION	NUMBER OF CASES	AGE ± SD (min-max age)	RACE OR ETHNICITY	GENDER (male%)	MISCELLANEOUS
**BDNF**	rs6265r	Failla [1]	Acute: no-risk allele highest mortality. Post-acute: no-risk group, young people lower risk: no-risk group old people higher risk	Mortality	Severe	-	GCS	Prospective, long	Acute 0-7d. Post-Acute 8d-1y	315	16-74	White	80%	
	rs7124442 and rs1519480	Rostami[2]	Impaired recovery especially at phase II	General cognitive intelligence		Penetrating injury	AFQT, MMSE, WMS-III, CT scan	Caveness (VHIS) Prospective, long-term follow-up study	10-15 and 30-35y post TBI	109	58.8 ± 2.8	White	100%	
	rs6265	Krueger [3]	Met allele but not the hypothesized Val allele promotes recovery on executive func, no difference overall	Executive recovery		Frontal lobe lesion group with focal penetrating head injuries	D-KEFS, WMS-III, AFQT-7A, CT	Prospective, long-term follow-up study	Chronic	168 Vietnam combat veterans	-	White	100%	
	rs6265	Siironen [4]	Increased risk	Disability and poor outcome		Subarachnoid hemorrhage	WFNS scale, GOS	Prospective, short-term	3m	105 survivors	47	Finns	50%	
	rs6265	Barbey [5]	Lower executive recovery	Executive function		Frontal lobe lesion group with focal penetrating head injuries	CT, WAIS III	VHIS phase 3	Chronic	156 Vietnam combat veterans	59	96% White	10%	
	rs6265	Narayanan [6]	Lower scores at both admission and follow-up	Neuropsychological function	Mild	-	GCS, MRI, (S-NAB Form 1)	Prospective, short-term	6m	48	27 ±8	Kuala Lumpur	87%	
	rs6265	Bagnato [7]	No association	Conscious-ness	TBI-induced vegetative state	-	LCF	Retrospective, multi-centric	1, 3, 6 and 12m	53	31 ±11 (15-55)	White (Mostly Italian)	84%	

	Rs6265	Gagner [8]	Initial protection that disappeared at 18m	Anxiety, depression	Mild			Prospective, longitudinal	6 and 16 m	145	1.5-5			
	rs7934165<br>rs6416056<br>rs4074134	Treble-Barna [9]	Lower recovery	General outcome	Mild to severe	-	BRIEF, CBCL	Prospective longitudinal	6, 12, 18m, 3.5y and 6y	135	3-7y	White 47%	52%	ACE, ADORA1, BDNF, IL1b, IL1RN, IL6, NT5E, and TNF
	rs1157659	Hayes [10]	Reduced functional connectivity	Hippocampal volume	Mild	-	mBAT,CAPS, SCID-IV, WTAR	Cross	years	165	310.4±8.2	White	92.7%	ApoE not relevant Other 8 irrelevant BDNF SNPs analyzed
**DRD1**	rs686	Pardini [11]	Greater aggression levels due to medial prefrontal cortex lesions but reduced aggression levels due to lateral prefrontal cortex	Aggression vs dopamine		Penetrating injury	CT, NPI-a, ETI	VHIS phase 3	36-39y	141 Vietnam war veterans, 29 Ctrl	-	White	100%	D2 rs4648317, and COMT Val158Met also analyzed
	rs3766553 and rs10920573	Wagner [12]	Increased risk	Time for first seizure	Severe	Non-penetrating trauma	GCS, CT, EEG	Retrospective	6y	206	34 ±1	-	80.20%	rs3766553, rs903361, rs10920573, rs6701725, rs17511192 analyzed
**DRD2**	rs6279	Failla [13]	Variant associated at 6 months, after that, no association	Cognition, depression, global outcome	Severe	Non-penetrating trauma	GCS, GOS, FIM-Cog, Trail making test, Digit span (WAIS-R), ROCFT, CVLTII, COWA,	Prospective	6, 12m	108	34.19 ±13.75 (7-71)	Mostly White (n=99)	81.50%	ANNK1 gene (rs1800497) and 6 tagging SNPs in the DRD2 gene (rs6279, rs2734838, rs17529477, rs4245147, rs7131056,

							DKEFS, Stroop Task, PHQ9							rs4630328) were genotyped
	rs6277	Yue [14]	T-allele better verbal learning and recall, NO for mental flexibility and non-verbal processing	Executive function	All grades	External force trauma	CVLT-II, WAIS-PSI, TMT, CT	TRACK-TBI Pilot	6m	128	44±16	White	64%	ANKK1 TaqIA (rs1800497) also analyzed
	rs6279	Myrga [15]	Independently associated with cognition at 6 months, with trends for a sex × gene interaction at 12 months	Verbal, attention, memory and executive	Severe	Non-penetrating trauma	Novel genetic risk score, WAIS-R, CVLT-II, TMT, DKEFS	Short-term	6 and 12m	193	34.7 ±13	White (182)	around 80%	ANKK1 (rs1800497, Taq1A variant), DRD2 (rs6279), COMT (rs4680), VMAT2 (also referred to as SLC18A2, rs363226), and DAT1 (also referred to as SLC6A3, variable number tandem repeat)
**ANKK1**	rs1800497 (Taq1A)	Failla [13]	Variant associated at 6 months, at 12 months to functional cognition, but no association after multiple comparisons	Cognition, depression, global outcome	Severe	Non-penetrating trauma	GCS, GOS, FIM-Cog, Trail making test, Digit span (WAIS-R), ROCFT, CVLTII, COWA, DKEFS, Stroop Task, PHQ9	Prospective	6, 12m	108	34.19 ±13.75 (7-71)	Mostly White (n=99)	81.50%	ANNK1 gene (rs1800497) and 6 tagging SNPs in the DRD2 gene (rs6279, rs2734838, rs17529477, rs4245147, rs7131056, rs4630328) were genotyped

	rs1800497 and rs2734849, association could be found only for short-term	Treble-Barna [16]	Lower neurobehavioral recovery	Executive function	All grades	-	BRIEF, CBCL, NINDS	Prospective longitudinal	Short and long-term. 3, 6, 12, 18m and 3.5 and 7y	TBI=68, Ctrl=72	(3-7)	81.9% White	51.40%	32 SNPs in dopamine-related genes, DRD2. SLC6A3, SLC18A2, COMT, and ANKK1 were also analyzed
	rs1800497	Yue [14]	T/T homozygotes scoring lowest	Verbal learning and non-verbal processing	All grades	Blunt trauma	CVLT-II, WAIS-PSI, GCS, SWLS, TMT, BSI18, GSI	2 prospective multicentric studies (COBRIT, TRACK-TBI)	6m	n=492: COBRIT n=272, TRACK-TBI Pilot n=220	40 ±16	White 76%, African-American/African 13%, other 11%	-	
	rs1800497 (Taq1A)	Myrga [17]	Associated with behavioral dysfunction after 12 months	Depression	Severe	Blunt trauma	FrSBe, PHQ9	Longitudinal	6, 12m	90	34 ±13	White	65%	
	rs1800497 (Taq1A)	Myrga [15]	Independently associated with cognition at 6 months, with trends for a sex × gene interaction at 12 months	Verbal, attention, memory and executive	Severe	Non-penetrating trauma	Novel genetic risk score, WAIS-R, CVLT-II, TMT, DKEFS	Short-term	6 and 12m	193	34.7 ±13	White (182)	around 80%	ANKK1 (rs1800497, Taq1A variant), DRD2 (rs6279), COMT (rs4680), VMAT2 (also referred to as SLC18A2, rs363226), and DAT1 (also referred to as SLC6A3, variable number tandem repeat)
	rs1800497 (Taq1A)	Wagner [12]	Lower caudate and putamen DAT binding among DRD2	Dopaminergic binding	Moderate/Severe	Non-penetrating trauma	GCS, MRI, PET	Prospective	1y	TBI=12, Ctrl=13	?	White	100%	Referred in the article as DRD2 Taq1a variant

			A2/A2 homozygotes											
**COMT**	rs4680	Winkler [18]	Met158 allele is associated with lower incidence of PTSD after univariate but NOT after multivariate analysis	PTSD	Mild	External force trauma	GOSE	TRACK-TBI Pilot	6m	93	40	White (70%), African American (14%), Asian (7%), mixed race (7%), American Indian/Native Alaskan (2%) or Hawaiian/Pacific Islander (2%)	60%	
	rs4680	Myrga [15]	Associated with behavioral dysfunction after 12 months	Depression	Severe	Blunt trauma	FrSBe, PHQ9	Longitudinal	6, 12m	90	34 ±13	White	65%	
	rs4680	Willmott [19]	No association	General outcome, PTA	Moderate/Severe	-	GCS, GOSE, Westmead PTA Scale, TMT, WAIS, RAVLT, RCFT, WTAR, CT	Prospective longitudinal long-term	up to 2y	223	36 ±16	White (94.2%), Asian (5.4%), African (0.4%)	71%	
	rs4680	Myrga [15]	Independently associated with cognition at 6 months, with trends for sex × gene interaction at 12 months	Verbal, attention, memory and executive	Severe	Non-penetrating trauma	Novel genetic risk score, WAIS-R, CVLT-II, TMT, DKEFS	Short-term	6 and 12m	193	34.7 ±13	White (182)	around 80%	ANKK1 (rs1800497, Taq1A variant), DRD2 (rs6279), COMT (rs4680), VMAT2 (also referred to as SLC18A2, rs363226), and DAT1 (also referred to as SLC6A3, variable number

														tandem repeat)
	rs4680	Nekrosius [20]	Increased risk for delirium only	Delirium, functional and cognitive	Mild to moderate	-	CAM, GODS MCAT	Prospective observational	4d	89	56	-	81%	
	Rs4680	Hayes [21]	Association	Decreased hippocampus and PTSD	Mild	-	CAPS, WTAR, SCID	Cross	years	146		White	90%	
**SLC18A2 (VMAT2)**	rs363226	Myrga [15]	Independently associated with cognition at 6 months, with trends for sex × gene interaction at 12 months	Verbal, attention, memory and executive	Severe	Non-penetrating trauma	Novel genetic risk score, WAIS-R, CVLT-II, TMT, DKEFS	Short-term	6 and 12m	193	34.7 ±13	White (182)	around 80%	ANKK1 (rs1800497, Taq1A variant), DRD2 (rs6279), COMT (rs4680), VMAT2 (also referred to as SLC18A2, rs363226), and DAT1 (also referred to as SLC6A3, variable number tandem repeat)
**SLC6A3 (DAT1)**	rs464049, rs460000	Treble-Barna [16]	Lower neurobehavioral recovery	Executive function	All grades	-	BRIEF, CBCL, NINDS	Prospective longitudinal	Short and long-term. 3, 6, 12, 18m and 3.5 and 7y	TBI=68, Ctrl=72	3-7	81.9% White	51.40%	32 SNPs in dopamine-related genes, DRD2. SLC6A3, SLC18A2, COMT, and ANKK1 were also analyzed

	rs1800497 (Taq1A)	Wagner [12]	Lower caudate and putamen DAT binding among DAT 9-allele carriers	Dopaminergic binding	Moderate/Severe	Non-penetrating trauma	GCS, MRI, PET	Prospective	1y	TBI=12, Ctrl=13	?	White	100%
**APOE**	E4	Miller [22]	Inconclusive	TPS	Severe	Non-penetrating trauma	GCS, EEG, GOS	Retrospective	6m	332	(18-75)	White	77%
	E4	Moran [23]	No association	Cognition	Mild	Concussion	CVLT-C, VMI, CANTAB, WASI, WRAT-3	Prospective longitudinal	1y	99	12	82% White	71%
	E4	Ponsford [24]	Low GOSE only in females	Cognition and PTA	All grades	-	GCS, GOSE, Westmead PTA Scale	Prospective	5y, mean 1.9	648	36 ±16	White 95.5%, 3.7% Asian, 0.8% African	67%
	E4	Yousuf [25]	No association	Overall recovery	All grades	-	GOS	Prospective	6m	450	40 ±14	Kashmiri	82%
	E4	Hiekkanen [26]	No association	PTA	All grades	-	MRI, GCS, GOSE, HICS	Prospective	1y	33	44 ±16	-	70%
	E4	Kassam [27]	Worse outcome	Overall recovery	All grades	-	GOS/GOSE	Meta-analysis, meta-regression	6m	358	(3-18)	-	variable
	E4	Treble-Barna [28]	Detrimental in positive environment, less optimal context non-carries is detrimental	Family environment and child functioning association	Moderate/Severe	-	GCS, CAFAS, BRIEF GEC, CBCL, PPQ, EA-HOME	Prospective longitudinal	6.8y	TBI=65, Ctrl=70	5 ±1	72% White	55%
	E4	Noe [29]	Inconclusive	PTA/memory	Moderate/Severe	-	CVLT, WAIS-III, GOAT	-	6m	189	31 ±15%	-	71%
	E4	Banks [30]	No association	Cognition and brain structure	All grades	Non-penetrating trauma	MRI and cognitive score	PFBHS	over 5y	193 pro fighters	30.5	Multiracial	around 92%
	E4	Merritt [31]	Worse outcome	Cognition, memory	Mild	mix	CVLT-II, WMS-IV, RCTF, WASI,	Cross sectional	76 m	99	32.4±6.37	58.6%	77.8%

						WAIS, D-KEFS, WCST,						
E4	Yue [32]	No early association<br><br>Worse outcome at 6 m	Verbal memory	Mild	Non-penetrating trauma	TRACK-TBI, CVLT-II, IRT, LDFR, LDCR, SDCR	Prospective	6m	114		White 73%	67%

Supplemental Table 1. Summary of impact of polymorphisms in widely studied genes associated with TBI on clinical outcomes.

Abbreviations. CAM, confusion assessment method; MCAST, Montreal cognitive Assessment test; GODS, Glasgow Outcome and Discharge Scale; CVLT-II, California Verbal Learning Test-Second Edition; WMS–IV, Wechsler Memory Scale–Fourth Edition; RCFT, Rey Complex Figure Test; WASI–II; Wechsler Abbreviated Scale of Intelligence–Second Edition; WAIS–IV, Wechsler Adult Intelligence Scale–Fourth Edition; D-KEFS, Delis–Kaplan Executive Function System; WCST, Wisconsin Card Sorting Test; CASP, Clinician-Administered PTSD Scale; WTAR, Wechsler Test of Adult Reading; SCID, Structured Clinical Interview for DMS-IV disorders; BAT, Boston Assessment of TBI-lifetime; AFQT, Armed Forces Qualification Test; MMSE, Mini Mental State Examination; Wechsler Memory Scale-III; VHIS, Vietnam Head Injury Study; D-KEFS, Delis-Kaplan Executive Function System; WFNS, World Federation on Neurological Societies; NAB, Neuropsychological Assessment Battery Screening; LCF, Level of Cognitive Function; NPI-a, neuropsychiatric inventory irritability and aggression; ETI, Emergent endotracheal intubation; PTSD, post-traumatic stress disorder; PTE, post-traumatic epilepsy; AB, amyloid beta plaque; GCS, Glasgow coma scale; CT, computational tomography; EEG, electroencephalogram; GOS, Glasgow outcome scale, WRAT, Wide range achievement test; CPT, cognitive processing therapy; CVLT, California verbal learning test; ISS, Injury severity score; APACHE, Acute physiology and chronic health evaluation; SAC, Standardized assessment of concussion; SCAT, Sport concussion assessment tool; TRACK-TBI, Transforming Research and Clinical Knowledge in Traumatic Brain Injury, TDA, Topological data analysis; WAIS, Weschler adult intelligence scale; ICP, intracranial pressure; DRS NRS-R ,Disability rating scale and neurobehavioral rating scale-revised; CAPS, Counselor-Assisted Problem Solving; TLEQ, Traumatic Life Events Questionnaire; Ctrl, control; CSF, cerebrospinal fluid; -, no data available. BRIEF, Behavior Rating Inventory of Executive Function; CBCL, Child Behavioral Check List; PUFA, Poly-unsaturated fatty acids.

1 Failla, M. D. *et al.* Variation in the BDNF gene interacts with age to predict mortality in a prospective, longitudinal cohort with severe TBI. *Neurorehabil Neural Repair* **29**, 234-246, doi:10.1177/1545968314542617 (2015).

2 Rostami, E. *et al.* BDNF polymorphism predicts general intelligence after penetrating traumatic brain injury. *PLoS One* **6**, e27389, doi:10.1371/journal.pone.0027389 (2011).

3 Krueger, F. *et al.* The role of the Met66 brain-derived neurotrophic factor allele in the recovery of executive functioning after combat-related traumatic brain injury. *J Neurosci* **31**, 598-606, doi:10.1523/JNEUROSCI.1399-10.2011 (2011).

4 Siironen, J. *et al.* The Met allele of the BDNF Val66Met polymorphism predicts poor outcome among survivors of aneurysmal subarachnoid hemorrhage. *Stroke* **38**, 2858-2860, doi:10.1161/STROKEAHA.107.485441 (2007).

5 Barbey, A. K. *et al.* Preservation of general intelligence following traumatic brain injury: contributions of the Met66 brain-derived neurotrophic factor. *PLoS One* **9**, e88733, doi:10.1371/journal.pone.0088733 (2014).

6 Narayanan, V. *et al.* Missense Mutation of Brain Derived Neurotrophic Factor (BDNF) Alters Neurocognitive Performance in Patients with Mild Traumatic Brain Injury: A Longitudinal Study. *PLoS One* **11**, e0158838, doi:10.1371/journal.pone.0158838 (2016).

7 Bagnato, S. *et al.* Brain-derived neurotrophic factor (Val66Met) polymorphism does not influence recovery from a post-traumatic vegetative state: a blinded retrospective multi-centric study. *J Neurotrauma* **29**, 2050-2059, doi:10.1089/neu.2011.2184 (2012).

8 Gagner, C., Tuerk, C. B., De Beaumont, L., Bernier, A. & Beauchamp, M. H. BDNF Val66Met polymorphism and internalizing behaviors after early mild traumatic brain injury. *J Neurotrauma*, doi:10.1089/neu.2019.6936 (2020).
9 Treble-Barna, A. *et al.* Cumulative Influence of Inflammatory Response Genetic Variation on Long-Term Neurobehavioral Outcomes after Pediatric Traumatic Brain Injury Relative to Orthopedic Injury: An Exploratory Polygenic Risk Score. *J Neurotrauma* **37**, 1491-1503, doi:10.1089/neu.2019.6866 (2020).
10 Hayes, J. P. *et al.* BDNF genotype is associated with hippocampal volume in mild traumatic brain injury. *Genes Brain Behav* **17**, 107-117, doi:10.1111/gbb.12403 (2018).
11 Pardini, M. *et al.* Aggression, DRD1 polymorphism, and lesion location in penetrating traumatic brain injury. *CNS Spectr* **19**, 382-390, doi:10.1017/S1092852914000108 (2014).
12 Wagner, A. K. *et al.* The influence of genetic variants on striatal dopamine transporter and D2 receptor binding after TBI. *J Cereb Blood Flow Metab* **34**, 1328-1339, doi:10.1038/jcbfm.2014.87 (2014).
13 Failla, M. D. *et al.* Posttraumatic Brain Injury Cognitive Performance Is Moderated by Variation Within ANKK1 and DRD2 Genes. *J Head Trauma Rehabil* **30**, E54-66, doi:10.1097/HTR.0000000000000118 (2015).
14 Yue, J. K. *et al.* DRD2 C957T polymorphism is associated with improved 6-month verbal learning following traumatic brain injury. *Neurogenetics* **18**, 29-38, doi:10.1007/s10048-016-0500-6 (2017).
15 Myrga, J. M. *et al.* A Dopamine Pathway Gene Risk Score for Cognitive Recovery Following Traumatic Brain Injury: Methodological Considerations, Preliminary Findings, and Interactions With Sex. *J Head Trauma Rehabil* **31**, E15-29, doi:10.1097/HTR.0000000000000199 (2016).
16 Treble-Barna, A. *et al.* Influence of Dopamine-Related Genes on Neurobehavioral Recovery after Traumatic Brain Injury during Early Childhood. *J Neurotrauma* **34**, 1919-1931, doi:10.1089/neu.2016.4840 (2017).
17 Myrga, J. M. *et al.* COMT and ANKK1 Genetics Interact With Depression to Influence Behavior Following Severe TBI: An Initial Assessment. *Neurorehabil Neural Repair* **30**, 920-930, doi:10.1177/1545968316648409 (2016).
18 Winkler, E. A. *et al.* COMT Val(158)Met polymorphism is associated with post-traumatic stress disorder and functional outcome following mild traumatic brain injury. *J Clin Neurosci* **35**, 109-116, doi:10.1016/j.jocn.2016.09.017 (2017).
19 Willmott, C., Withiel, T., Ponsford, J. & Burke, R. COMT Val158Met and cognitive and functional outcomes after traumatic brain injury. *J Neurotrauma* **31**, 1507-1514, doi:10.1089/neu.2013.3308 (2014).
20 Nekrosius, D. *et al.* Association of COMT Val(158)Met Polymorphism With Delirium Risk and Outcomes After Traumatic Brain Injury. *J Neuropsychiatry Clin Neurosci* **31**, 298-305, doi:10.1176/appi.neuropsych.18080195 (2019).
21 Hayes, J. P. *et al.* COMT Val158Met polymorphism moderates the association between PTSD symptom severity and hippocampal volume. *J Psychiatry Neurosci* **42**, 95-102, doi:10.1503/jpn.150339 (2017).
22 Miller, M. A. *et al.* APOE genetic associations with seizure development after severe traumatic brain injury. *Brain Inj* **24**, 1468-1477, doi:10.3109/02699052.2010.520299 (2010).
23 Moran, L. M. *et al.* Apolipoprotein E4 as a predictor of outcomes in pediatric mild traumatic brain injury. *J Neurotrauma* **26**, 1489-1495, doi:10.1089/neu.2008.0767 (2009).
24 Ponsford, J. *et al.* The association between apolipoprotein E and traumatic brain injury severity and functional outcome in a rehabilitation sample. *J Neurotrauma* **28**, 1683-1692, doi:10.1089/neu.2010.1623 (2011).
25 Yousuf, A. *et al.* Genetic Variation of ApoE Gene in Ethnic Kashmiri Population and Its Association with Outcome After Traumatic Brain Injury. *J Mol Neurosci* **56**, 597-601, doi:10.1007/s12031-015-0554-1 (2015).

26 Hiekkanen, H., Kurki, T., Brandstack, N., Kairisto, V. & Tenovuo, O. Association of injury severity, MRI-results and ApoE genotype with 1-year outcome in mainly mild TBI: a preliminary study. *Brain Inj* **23**, 396-402, doi:10.1080/02699050902926259 (2009).

27 Kassam, I., Gagnon, F. & Cusimano, M. D. Association of the APOE-epsilon4 allele with outcome of traumatic brain injury in children and youth: a meta-analysis and meta-regression. *J Neurol Neurosurg Psychiatry* **87**, 433-440, doi:10.1136/jnnp-2015-310500 (2016).

28 Treble-Barna, A. *et al.* Does Apolipoprotein e4 Status Moderate the Association of Family Environment with Long-Term Child Functioning following Early Moderate to Severe Traumatic Brain Injury? A Preliminary Study. *J Int Neuropsychol Soc* **22**, 859-864, doi:10.1017/S1355617716000631 (2016).

29 Noe, E., Ferri, J., Colomer, C., Moliner, B. & Chirivella, J. APOE genotype and verbal memory recovery during and after emergence from post-traumatic amnesia. *Brain Inj* **24**, 886-892, doi:10.3109/02699051003724952 (2010).

30 Banks, S. J., Miller, J. B., Rissman, R. A. & Bernick, C. B. Lack of Influence of Apolipoprotein E Status on Cognition or Brain Structure in Professional Fighters. *J Neurotrauma* **34**, 380-384, doi:10.1089/neu.2016.4453 (2017).

31 Merritt, V. C. *et al.* Apolipoprotein E (APOE) epsilon4 genotype is associated with reduced neuropsychological performance in military veterans with a history of mild traumatic brain injury. *J Clin Exp Neuropsychol* **40**, 1050-1061, doi:10.1080/13803395.2018.1508555 (2018).

32 Yue, J. K. *et al.* Apolipoprotein E epsilon 4 (APOE-epsilon4) genotype is associated with decreased 6-month verbal memory performance after mild traumatic brain injury. *Brain Behav* **7**, e00791, doi:10.1002/brb3.791 (2017).

GENE	SNP	AUTHOR	OUTCOME	FUNCTION ANALYZED	TBI GRADE (GCS)	NATURE OF LESION	ASSESMENT	TYPE OF STUDY	TIMEFRAME OF EVALUATION	NUMBER OF CASES	AGE ± SD (min-max age)	RACE OR ETHNICITY	GENDER (male%)	MISCELLANEOUS
**SLC1A1 (Glut transp)**	rs10974620 and rs7858819	Ritter [1]	Increased risk	PTSD	Severe	Non-penetrating trauma	GCS, CT, EEG	Prospective long-term	3y	253	35	White	79%	Thirty-two tagging SNPs were examined (SLC1A1: n = 28, SLC1A6: n = 4
**SCL1A3**	rs4869682	Kumar [2]	Increased risk	PTE	Severe	Non-penetrating trauma		Prospective	Up to 3y		35.2	White	78.4	SLC1A2: n=21, SLC1A3: n=18
**GAD1**	rs3828275, rs3791878, and rs769391	Darrah [3]	Increased risk	Time for first seizure	Severe	Non-penetrating trauma	GCS, EEG, GOS, CT	Retrospective	6m	257	35	White	78%	17 haplotypes tested
**ADK**	rs11001109	Diamond [4]	Shorter time to first seizure and an increased seizure rate 3y post-TBI	PTE	Moderate/Severe	Non-penetrating trauma	GCS, CT, EEG	Prospective longitudinal	Up to 3y	161	33 ±1	White	80%	Nine ADK, three CD73, and two ENT-1 tSNPs were analyzed
**NT5E (CD73)**	rs9444348	Diamond [4]	Shorter time to first seizure and an increased seizure rate 3y post-TBI	PTE	Moderate/Severe	Non-penetrating trauma	GCS, CT, EEG	Prospective longitudinal	Up to 3y	161	33 ±1	White	80%	Nine ADK, three CD73, and two ENT-1 tSNPs were analyzed
	rs6942065	Treble-Barna [5]	Lower recovery	General outcome	Mild to severe	BRIEF, CBCL	Prospective longitudinal	6, 12, 18m, 3.5y and 6y	135	3-7y	White	47%	52%	ACE, ADORA1, BDNF, IL1b, IL1RN, IL6, NT5E, and TNF
**ADORA 1**	rs3766553 and rs10920573	Wagner [6]	Association	PTSD	Severe	-	GCS, EEG, GOS	Prospective	6y	206	34.3 ± 1	White	80%	rs3766553, rs903361, rs10920573, rs6701725, and rs17511192 analyzed
**MME, CALLA, Neprilysin**	GT repeats	Johnson [7]	Increased risk	AB plaque deposition after TBI	Severe	-	-	Histological	post-mortem	90	33.2	European White	74.00%	
**SNCA**	rs1372525	Shee [8]	Protective association in memory for the A minor allele	Intellectual function, attentional function, and memory	Mild	-	GCS, WRAT, CPT, CVLT	Short term, transversal	1m	91 patients, 81 ctrl	TBI=33.7 ±13.7, Ctrl=47.9 ±10.2	95% White	TBI=61%, Ctrl=31%	13 SNCA SNPs analyzed

**IL-1**	rs315919<br><br>rs3213448	Treble-Barna [5]	Lower recovery	General outcome	Mild to severe	-	BRIEF, CBCL	Prospective longitudinal	6, 12, 18m, 3.5y and 6y	135	3-7y	White 47%	52%	ACE, ADORA1, BDNF, IL1b, IL1RN, IL6, NT5E, and TNF
**IL-1b**	rs1143634	Cotter [9]	Increased risk	PTE		-	-	Meta-analysis		-	-	-	-	
	rs1143634	Diamond [10]	Increased risk	PTE	Moderate/Severe	-	Time for the first seizure, EEG, ISS	Longitudinal retrospective	1w to 1y	256	35	White	81.60%	5 SNPs within the gene were analyzed
**Il-6**	rs1800795	Dalla [11]	Increased risk	Death	Severe	-	GCS, GOS, APACHE	Short-term	Acute	77	-	White	100.00%	promoter region
**IL-6R**	rs22281450	Terrel [12]	Increased risk	Risk of concussion	All grades	Non-penetrating trauma	SAC, SCAT	Multicenter prospective	4y	1057	20 ±1	59.4% White, 35.0% African-American, 5.6% other	189.30%	APOE, APOE G-219T promoter, microtubule associated protein (MAPT)/tau exon 6 Ser53Pro, MAPT/tau Hist47Tyr, IL-6572 G/C and IL-6RAsp358Ala
**MTHFR**	C677T	Cotter [9]	Increased risk	PTE	All grades	-	Reported epilepsy	Meta-analysis	--	1600 military personnel	-	White (68%), black (19%), and other races (13%	80%	
**PARP**	rs3219119	Sarnaik [13]	Better outcome	Overall recovery	Severe	Non-penetrating trauma	GOS	Retrospective	6m	191	31.5	94.1% White and 4.3% African-American	79%	rs1109032, rs3219090, rs3219119, and rs2271347 were analyzed
	rs3219119	Nielson [14]	Increased risk	Predictor of outcome	All grades	Non-penetrating trauma	TRAC-TBI, TDA, CT, PTSD test, WAIS, CVLT	Multicenter, longitudinal	6m	556	43.3±18.5	White (71.5%)	71.50%	ANKK1 (rs1800497) (rs4938016) (rs11604671), COMT (rs4680), DRD2 (rs6277,), BDNF (rs6265), 5HT2A receptor (rs6311), ApoE-ε2 (rs7412) and ApoE-ε4 (rs429358), OPRM1 (rs1799971), BCL2 (rs17759659), PARP-1 (rs3219119).
**ABCC8**	rs2283261, rs3819521, rs2283258, rs1799857	Jha [15]	4 minor alleles at risk, major rs1799857 preventive	Cerebral edema	Severe	Non-penetrating trauma	CT, ICP	Prospective	Acute	385	37.9 ±16.8	White (98.7%)	79%	14 SNPs identified
**PPP3CC**	rs2443504	Osier [16]	Increased risk and mortality	General outcome	Severe	-	GOS	Retrospective	3, 6, 12m	380	39.9 ±16	White	79%	rs10108011, rs2461491 and rs2469749 also analyzed
**Ngb**	rs3783988	Chuang [17]	Increased risk	General outcome	Severe	-	GOS, DRS NRS-R	Prospective	3, 6, 12, and 24m	196	34±14	White	78.60%	rs10133981

**AQP4**	rs3763043, rs3875089	Dardiotis [18]	rs3763043 higher risk, rs3875089 protective	General outcome	All grades	blunt trauma	GCS, GOS	Prospective	6m	363	42 ±21	Greek	80.40%	7 SNPs within the gene were analyzed
**BCL2**	rs17759659, rs1801018, rs7236090, and rs949037	Hoh [19]	Association	General outcome, cognition	Severe	Non-penetrating trauma	GOS, DRS NRS-R	Prospective	3, 6, 12, and 24m	205	34 ±14	White	79.50%	17 SNPs analyzed
	Rs17759659	Deng [20]	Increased risk	ICP	Severe		ICP<br>Edema<br>Surgery	Prospective observational	Acute	263	39.2	-	78%	
**Lectin Pathway genes**	MBL2 rs1800451, rs1800450, rs5030737, rs7096206; FCN2 rs3124953, rs17514136, rs17549193, rs7851696	Osthoff [21]	No association	Mortality, consciousness and general outcome	Severe	Non-penetrating trauma	-	Prospective, observational	90d	44	39	White 95%	75%	
**miR-431-3P**	rt11851174	You [22]	Association	Mortality, consciousness	Severe	Non-penetrating trauma	-	-	Short-term	TBI=26, Ctrl=21	50 ±8	Chinese	57%	
**MT DNA**	K haplotype	Bulstrode [23]	Better outcome	General outcome	-	-	GOS, CT	Prospective	6m	1094	35	Scottish	81%	5 haplotypes studied
**ND3**	A10398G	Conley [24]	Worse outcome	General outcome, cognition	Severe	Non-penetrating trauma	GOS, DRS NRS	Prospective	3, 6 and 12m	136	(16-80)	White	-	19 SNPs analyzed
**S100B**	rs1051169<br>rs9984765	Osier [25]	Better and worse outcome respectively	General outcome	Severe	Non-penetrating trauma	GOS	Prospective	3, 6, 12, 24 m	305	39 ±16.4	White	80.7%	18 SNPs analyzed including other genes (GFAP and UCHL1)
**C reactive Protein**	rs3091244<br>rs1205<br>rs2794520	Miller [26]	Increased severity of PTS	PTSD	-	-	CAPS, TLEQ	-	-	286	32 ±8.354	White (72.8%), Latino (15.4%), Black (8.6%), Asian (2.2%) American Indian (1.1%)	88.5%	AIM2m locus methylations was also associated

**ABCG2**	rs2231142	Adams [27]	Better score	General outcome	Severe	Non-penetrating trauma	GOS,<br>Imaging	Prospective longitudinal, two cohorts (Discovery and replication)	3, 6, 12, 24 m	270 and 166	33 (23–47)<br>36.5 (24–54)	White	78%	Negative interaction with age
**Aromatase**<br>**[CYP]19A1**	rs2470152, rs4646,<br>rs2470144	Garringer [28]	Worse score	Mortality<br>General outcome	Severe	Non-penetrating trauma	GOS<br>Hormone levels	Prospective longitudinal	6 m	110	34.8 ± 1.5	White	81.8%	18 SNPs evaluated<br>Higher CSF estradiol better outcome
**FADS1**	rs174537	Waits [29]	High association<br>Worse outcome	General outcome	-	-	PUFA, Inflammatory cytokines	prospective, single-center, observational pilot	Acute	130		White	-	
**P53**	rs1042522	Mellet [30]	Worse outcome	General outcome	severe	Non-penetrating trauma	GCS, GOS, NRS, DRS	Prospective	24 m	429	37.4	78.3%	85.5%	
**ACE**	rs4362<br>rs4329	Treble-Barna [5]	Lower recovery	General outcome	Mild to severe	-	BRIEF, CBCL	Prospective longitudinal	6, 12, 18m, 3.5y and 6y	135	3-7y	White 47%	52%	ACE, ADORA1, BDNF, IL1b, IL1RN, IL6, NT5E, and TNF

Supplemental Table 2. Summary of the impact of some less widely studied genetic polymorphisms associated with recovery from TBI on clinical outcomes

Abbreviations, PTSD, post-traumatic stress disorder; PTE, post-traumatic epilepsy; AB, amyloid beta plaque; GCS, Glasgow coma scale; CT, computational tomography; EEG, electroencephalogram; GOS, Glasgow outcome scale, WRAT, Wide range achievement test; CPT, cognitive processing therapy; CVLT, California verbal learning test; ISS, Injury severity score; APACHE, Acute physiology and chronic health evaluation; SAC, Standardized assessment of concussion; SCAT, Sport concussion assessment tool; TRACK-TBI, Transforming Research and Clinical Knowledge in Traumatic Brain Injury, TDA, Topological data analysis; WAIS, Weschler adult intelligence scale; ICP, intracranial pressure; DRS NRS-R ,Disability rating scale and neurobehavioral rating scale-revised; CAPS, Counselor-Assisted Problem Solving; TLEQ, Traumatic Life Events Questionnaire; Ctrl, control; CSF, cerebrospinal fluid; -, no data available. BRIEF, Behavior Rating Inventory of Executive Function; CBCL, Child Behavioral Check List; PUFA, Poly-unsaturated fatty acid.

1 Ritter, A. C., Kammerer, C. M., Brooks, M. M., Conley, Y. P. & Wagner, A. K. Genetic variation in neuronal glutamate transport genes and associations with posttraumatic seizure. *Epilepsia* **57**, 984-993, doi:10.1111/epi.13397 (2016).
2 Kumar, R. G., Breslin, K. B., Ritter, A. C., Conley, Y. P. & Wagner, A. K. Variability with Astroglial Glutamate Transport Genetics Is Associated with Increased Risk for Post-Traumatic Seizures. *J Neurotrauma* **36**, 230-238, doi:10.1089/neu.2018.5632 (2019).
3 Darrah, S. D. *et al.* Genetic variability in glutamic acid decarboxylase genes: associations with post-traumatic seizures after severe TBI. *Epilepsy Res* **103**, 180-194, doi:10.1016/j.eplepsyres.2012.07.006 (2013).
4 Diamond, M. L. *et al.* Genetic variation in the adenosine regulatory cycle is associated with posttraumatic epilepsy development. *Epilepsia* **56**, 1198-1206, doi:10.1111/epi.13044 (2015).
5 Treble-Barna, A. *et al.* Cumulative Influence of Inflammatory Response Genetic Variation on Long-Term Neurobehavioral Outcomes after Pediatric Traumatic Brain Injury Relative to Orthopedic Injury: An Exploratory Polygenic Risk Score. *J Neurotrauma* **37**, 1491-1503, doi:10.1089/neu.2019.6866 (2020).
6 Wagner, A. K. *et al.* Adenosine A1 receptor gene variants associated with post-traumatic seizures after severe TBI. *Epilepsy Res* **90**, 259-272, doi:10.1016/j.eplepsyres.2010.06.001 (2010).
7 Johnson, V. E. *et al.* A neprilysin polymorphism and amyloid-beta plaques after traumatic brain injury. *J Neurotrauma* **26**, 1197-1202, doi:10.1089/neu.2008-0843

10.1089/neu.2008.0843 (2009).
8 Shee, K. *et al.* Alpha-synuclein (SNCA) polymorphisms exert protective effects on memory after mild traumatic brain injury. *Neurosci Lett* **630**, 241-246, doi:10.1016/j.neulet.2016.07.057 (2016).
9 Cotter, D., Kelso, A. & Neligan, A. Genetic biomarkers of posttraumatic epilepsy: A systematic review. *Seizure* **46**, 53-58, doi:10.1016/j.seizure.2017.02.002 (2017).
10 Diamond, M. L. *et al.* IL-1beta associations with posttraumatic epilepsy development: a genetics and biomarker cohort study. *Epilepsia* **55**, 1109-1119, doi:10.1111/epi.12628 (2014).
11 Dalla Libera, A. L. *et al.* IL-6 polymorphism associated with fatal outcome in patients with severe traumatic brain injury. *Brain Inj* **25**, 365-369, doi:10.3109/02699052.2011.556107 (2011).
12 Terrell, T. R. *et al.* Genetic polymorphisms associated with the risk of concussion in 1056 college athletes: a multicentre prospective cohort study. *Br J Sports Med* **52**, 192-198, doi:10.1136/bjsports-2016-097419 (2018).
13 Sarnaik, A. A. *et al.* Influence of PARP-1 polymorphisms in patients after traumatic brain injury. *J Neurotrauma* **27**, 465-471, doi:10.1089/neu.2009.1171 (2010).
14 Nielson, J. L. *et al.* Uncovering precision phenotype-biomarker associations in traumatic brain injury using topological data analysis. *PLoS One* **12**, e0169490, doi:10.1371/journal.pone.0169490 (2017).
15 Jha, R. M. *et al.* ABCC8 Single Nucleotide Polymorphisms are Associated with Cerebral Edema in Severe TBI. *Neurocrit Care* **26**, 213-224, doi:10.1007/s12028-016-0309-z (2017).
16 Osier, N. D. *et al.* Variation in PPP3CC Genotype Is Associated with Long-Term Recovery after Severe Brain Injury. *J Neurotrauma* **34**, 86-96, doi:10.1089/neu.2015.4343 (2017).

17 Chuang, P. Y. *et al.* Neuroglobin genetic polymorphisms and their relationship to functional outcomes after traumatic brain injury. *J Neurotrauma* **27**, 999-1006, doi:10.1089/neu.2009.1129 (2010).

18 Dardiotis, E. *et al.* AQP4 tag single nucleotide polymorphisms in patients with traumatic brain injury. *J Neurotrauma* **31**, 1920-1926, doi:10.1089/neu.2014.3347 (2014).

19 Hoh, N. Z. *et al.* BCL2 genotypes: functional and neurobehavioral outcomes after severe traumatic brain injury. *J Neurotrauma* **27**, 1413-1427, doi:10.1089/neu.2009.1256 (2010).

20 Deng, H. *et al.* B-cell lymphoma 2 (Bcl-2) gene is associated with intracranial hypertension after severe traumatic brain injury. *J Neurotrauma*, doi:10.1089/neu.2020.7028 (2020).

21 Osthoff, M., Walder, B., Delhumeau, C., Trendelenburg, M. & Turck, N. Association of Lectin Pathway Protein Levels and Genetic Variants Early after Injury with Outcomes after Severe Traumatic Brain Injury: A Prospective Cohort Study. *J Neurotrauma* **34**, 2560-2566, doi:10.1089/neu.2016.4941 (2017).

22 You, W. D. *et al.* Alteration of microRNA expression in cerebrospinal fluid of unconscious patients after traumatic brain injury and a bioinformatic analysis of related single nucleotide polymorphisms. *Chin J Traumatol* **19**, 11-15, doi:10.1016/j.cjtee.2016.01.004 (2016).

23 Bulstrode, H. *et al.* Mitochondrial DNA and traumatic brain injury. *Ann Neurol* **75**, 186-195, doi:10.1002/ana.24116 (2014).

24 Conley, Y. P. *et al.* Mitochondrial polymorphisms impact outcomes after severe traumatic brain injury. *J Neurotrauma* **31**, 34-41, doi:10.1089/neu.2013.2855 (2014).

25 Osier, N. D., Conley, Y. P., Okonkwo, D. O. & Puccio, A. M. Variation in Candidate Traumatic Brain Injury Biomarker Genes Are Associated with Gross Neurological Outcomes after Severe Traumatic Brain Injury. *J Neurotrauma* **35**, 2684-2690, doi:10.1089/neu.2017.5268 (2018).

26 Miller, M. W. *et al.* CRP polymorphisms and DNA methylation of the AIM2 gene influence associations between trauma exposure, PTSD, and C-reactive protein. *Brain Behav Immun* **67**, 194-202, doi:10.1016/j.bbi.2017.08.022 (2018).

27 Adams, S. M. *et al.* ABCG2 c.421C>A Is Associated with Outcomes after Severe Traumatic Brain Injury. *J Neurotrauma* **35**, 48-53, doi:10.1089/neu.2017.5000 (2018).

28 Garringer, J. A. *et al.* Impact of aromatase genetic variation on hormone levels and global outcome after severe TBI. *J Neurotrauma* **30**, 1415-1425, doi:10.1089/neu.2012.2565 (2013).

29 Waits, C. M. K. *et al.* A Pilot Study Assessing the Impact of rs174537 on Circulating Polyunsaturated Fatty Acids and the Inflammatory Response in Patients with Traumatic Brain Injury. *J Neurotrauma*, doi:10.1089/neu.2019.6734 (2020).

30 Mellett, K. *et al.* Genetic Variation in the TP53 Gene and Patient Outcomes Following Severe Traumatic Brain Injury. *Biol Res Nurs* **22**, 334-340, doi:10.1177/1099800420912335 (2020).
